# *Mazus unguiculatus* (Mazaceae), a distinct new species from Anhui Province, eastern China

**DOI:** 10.3897/phytokeys.274.183749

**Published:** 2026-04-24

**Authors:** Si-Cheng Xu, Dao-Zhang Min, Bo Li

**Affiliations:** 1 Center for Integrative Conservation, Xishuangbanna Tropical Botanical Garden, Chinese Academy of Sciences, Mengla 666303, China Xinjiang Institute of Ecology and Geography, Chinese Academy of Sciences Urumqi China; 2 University of Chinese Academy of Sciences, Beijing 100049, China Xishuangbanna Tropical Botanical Garden, Chinese Academy of Sciences Mengla China; 3 State Key Laboratory of Ecological Safety and Sustainable Development in Arid Lands, Xinjiang Institute of Ecology and Geography, Chinese Academy of Sciences, Urumqi 830011, China University of Chinese Academy of Sciences Beijing China; 4 Southeast Asia Biodiversity Research Institute, Chinese Academy of Sciences, Mengla 666303, China Southeast Asia Biodiversity Research Institute, Chinese Academy of Sciences Mengla China

**Keywords:** Lamiales, Mazaceae, morphology, new taxa, phylogeny

## Abstract

*Mazus unguiculatus* Bo Li, a new species of Mazaceae from Anhui Province in eastern China, is described and illustrated. Phylogenetic analyses based on combined chloroplast DNA regions (*matK*, *rbc*L, *rps*16, and *trn*L-F) and the nuclear ribosomal internal transcribed spacer (nrITS), together with morphological evidence, confirm that the new species is sister to *M. spicatus* Vaniot. It is distinguished from the latter and all other congeners by its unique corolla morphology, viz., the upper lip is bilobed, each lobe has an emarginate to bilobed apex, whereas the lower lip is deeply trilobed with subequal lobes; each lobe possesses a short claw at the base and shallow teeth at the middle of the apex, and the palate is absent. A detailed description is provided, along with information on its distribution, habitat, and conservation status.

## Introduction

Mazaceae is a recently established family within the order Lamiales, comprising approximately 50 species distributed across four genera, viz., *Dodartia* L., *Lancea* Hook.f. & Thomson, *Mazus* Lour., and *Puchiumazus* Bo Li, D.G.Zhang & C.L.Xiang (Xiang et al. 2021; Li et al. 2022; Plants of the World Online [POWO] 2025). Among these, *Mazus* is the largest genus, with approximately 45 species, primarily found in temperate and subtropical regions of Asia, as well as parts of Australia and New Zealand (Li 1954; Hong et al. 1998; POWO 2025). The genus is characterized by small herbaceous plants, a distinctly two-lipped corolla that usually forms a raised palate with two longitudinal plaits, and a capsule enclosed within the persistent calyx (Fischer 2004; Deng et al. 2019; Li et al. 2022). China represents the center of diversity for *Mazus*, hosting more than 30 species (Hong 1998; Chen et al. 2024). While most species are narrow endemics, a few, such as *M. pumilus* (Burm.f.) Steenis, exhibit a wide distribution across Eurasia and the Americas (POWO 2025).

The taxonomic placement and infrageneric classification of *Mazus* have undergone several revisions. Historically, it was placed in the subtribe Mimulinae (tribe Gratioleae) of Scrophulariaceae (von Wettstein 1891; Thieret 1954, 1967). Molecular phylogenetic studies later transferred *Mazus* to Phrymaceae, where it formed a clade with *Lancea* (Beardsley and Olmstead 2002; Oxelman et al. 2005; Albach et al. 2009; Schäferhoff et al. 2010). This clade was subsequently elevated to familial rank as Mazaceae, with the inclusion of *Mazus*, *Dodartia*, and *Lancea* (Reveal 2011). Within the genus, early classifications recognized two sections based on the presence or absence of stolons (Bonati 1908; Li 1954). This was later expanded to three sections, including *M.* sect. *Mazus*, *M.* sect. *Trichogynus* Tsoong, and *M.* sect. *Lanceifoliae* Bonati (Hong et al. 1998; Hsieh 2000). The last was recently segregated as the monotypic genus *Puchiumazus* (Xiang et al. 2021). Based on molecular and biogeographic evidence, Deng et al. (2019) proposed a revised infrageneric classification with two subgenera, the Asian *M.* subg. *Mazus* and the Australian *M.* subg. *Notomazus* T.Deng, N.Lin & H.Sun.

Species delimitation within *Mazus* has also been problematic due to high morphological variability (Li 1954). While Bonati (1908) recognized over 30 species, Li (1954) considered this treatment unnatural and later recognized only ca. 10 species (Li 1978). Royen (1983) instead recognized over 50 species. Hong et al. (1998) accepted approximately 35 species globally, including 25 in China. In recent years, taxonomic discovery of *Mazus* has accelerated significantly, with 10 new species described from China between 2016 and 2024 (Deng et al. 2016; Xiang et al. 2021; Li et al. 2022; Ju et al. 2023; Chen et al. 2024). These include five from Taiwan (Ying 2022, 2024), two from Hubei (*M. fruticosus* Bo Li, D.G.Zhang & C.L.Xiang and *M. sunhangii* D.G.Zhang & T.Deng), and one each from Fujian (*M. jiangshiensis* Y.Bin Chen, Xin Y.Chen & Liang Ma), Jiangxi (*M. danxiacola* Bo Li & B.Chen), and Xizang (*M. motuoensis* W.B.Ju, Bo Xu & X.F.Gao), respectively. These findings indicate a significant hidden diversity of *Mazus* in China.

**Figure 1.**
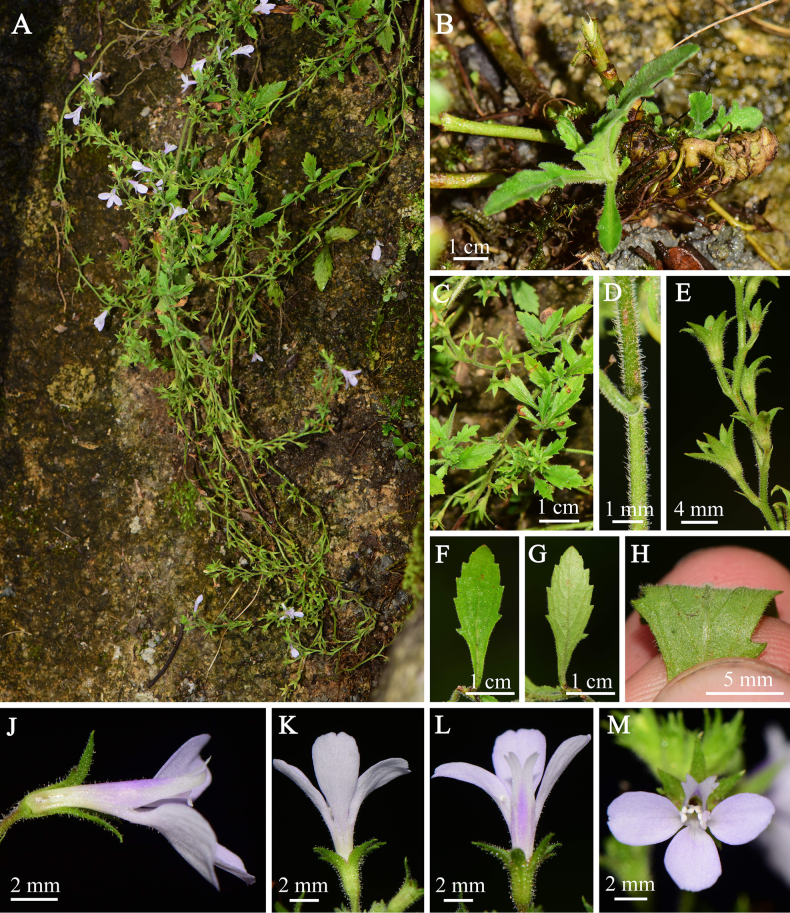
*Mazus unguiculatus* sp. nov. **A**. Habit; **B**. Basal leaves; **C**. Stem leaves; **D**. Stem, showing the indumentum; **E**. A portion of the inflorescence, showing persistent calyces; **F**. Leaf blade (adaxial view); **G**. Leaf blade (abaxial view); **H**. Abaxial surface of the leaf, showing indumentum and venation; **J–M**. Corolla (**J**. Lateral view; **K**. Basal view; **L**. Apical view; **M**. Frontal view). (Photographed by Xin-Xin Zhu).

**Figure 2.**
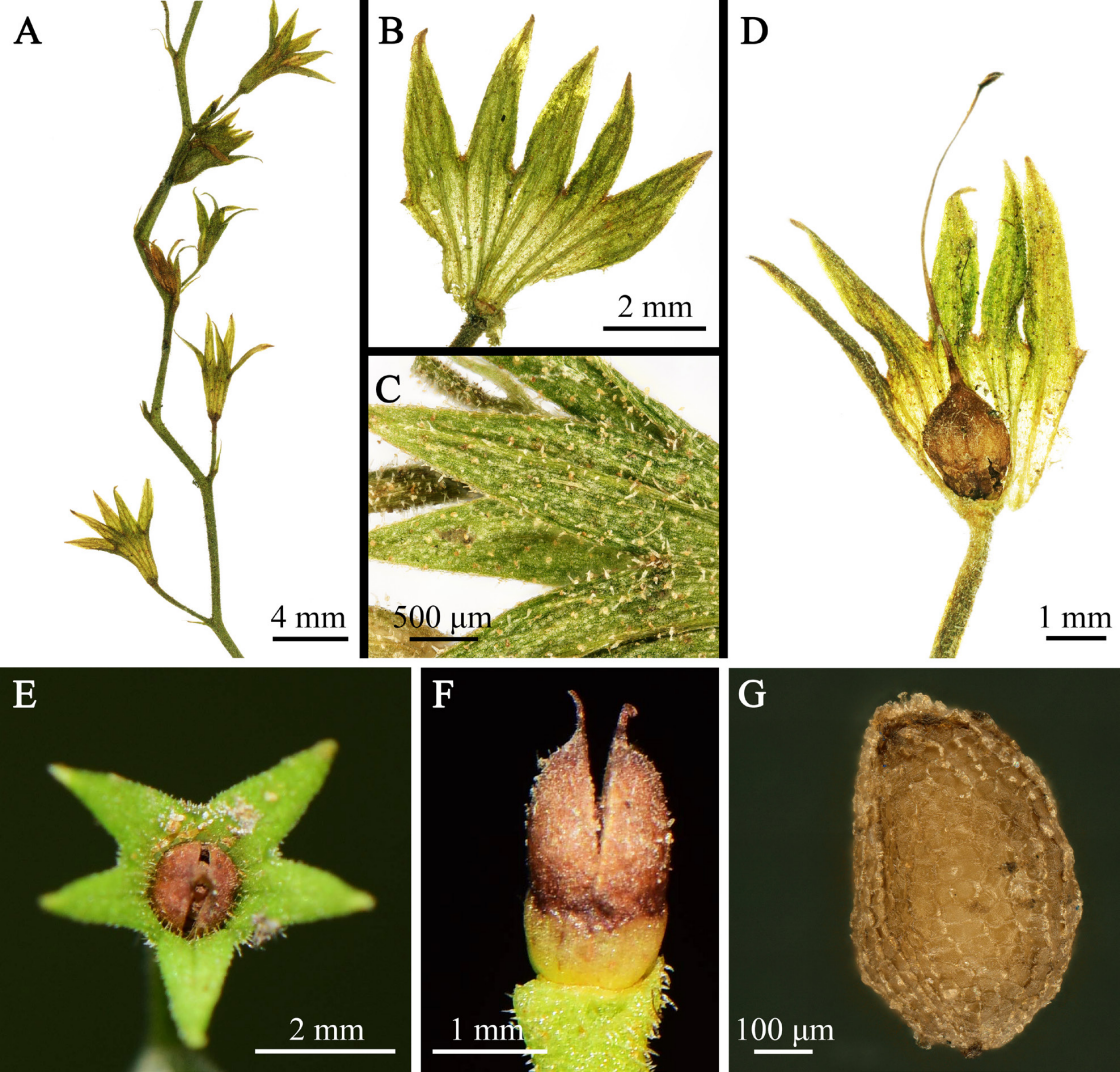
*Mazus unguiculatus* sp. nov. **A**. A portion of the inflorescence, showing persistent calyces; **B**. Dissected calyx, showing the 10 veins; **C**. Close-up of the calyx, showing the indumentum; **D**. Ovary; **E**. Capsule enclosed within the persistent calyx (apical view); **F**. Capsule (lateral view); **G**. Seed (lateral view). (**A–D, G**. Photographed by Bo Li; **E–F**. By Xin-Xin Zhu).

In July 2021, an unusual population of *Mazus* was discovered growing on steep rock crevices in Tianzhushan Mountain of Qianshan County, Anhui Province, eastern China. Morphologically, it closely resembles *M. spicatus* Vaniot in its white to pale rusty pubescence and hairy ovaries (Figs 1D, 2D). However, the new population can be readily distinguished by its weak, pendulous branches, markedly elongated inflorescences (Fig. 1A), and a unique corolla with lobes of the upper lip emarginate to bilobed and the lower lip deeply trilobed with subequal lobes, each forming a short claw at the base and shallow teeth at the apex, and the absence of a palate (Fig. 1K–M). Integrating morphological and molecular phylogenetic evidence, we here recognize it as a new species, *Mazus unguiculatus* Bo Li.

## Materials and methods

### Morphological study

Field surveys were conducted at Tianzhushan Mountain of Qianshan County, Anqing City, Anhui Province. Voucher specimens were collected and deposited in the Herbarium of Xishuangbanna Tropical Botanical Garden, Chinese Academy of Sciences (**HITBC**), and the Herbarium of Kunming Institute of Botany, Chinese Academy of Sciences (**KUN**). The morphology of the putative new species was examined based on living individuals and herbarium specimens. Measurements were taken under a stereo dissecting microscope using a ruler and a micrometer. Detailed morphological comparisons with *M. spicatus* were based on specimens deposited in CSFI, GZTM, HGAS, HIB, IBSC, KUN, NAS, PE, SM, WUK, and XBGH (acronyms according to Thiers 2025), as well as digital images of living plants. Relevant taxonomic and floristic literature of *Mazus* was reviewed. Terminology of morphological description follows Beentje (2016) and Hong et al. (1998).

### Molecular phylogenetic analysis

The systematic placement and relationships of *M. unguiculatus* were examined using Maximum Likelihood (ML) and Bayesian Inference (BI). Four plastid markers (*matK*, *rbc*L, *rps*16, and *trn*L-F) and the nuclear ribosomal internal transcribed spacer (nrITS) were used for phylogenetic reconstruction. Twenty-five *Mazus* species were sampled as ingroups of the cpDNA dataset, and 20 of them were selected for the nrITS dataset. Two species, *Dodartia orientalis* L. and *Lancea tibetica* Hook.f. & Thomson were selected as outgroups according to previous studies (Deng et al. 2019; Xiang et al. 2021). One individual of the potential new species (*Bo Li LB1093*), collected from its type locality, was newly sequenced, while all remaining sequences were retrieved from GenBank (https://www.ncbi.nlm.nih.gov). Voucher information and GenBank accession numbers are provided in Table 1. Procedures of DNA extraction, amplification, sequencing, and detailed settings of phylogenetic analyses followed Deng et al. (2019), Xiang et al. (2021), and Li et al. (2022).

**Table 1.** Taxa, GenBank accession numbers, and their vouchers used in this study. Herbarium abbreviations are listed after the vouchers. Newly sequenced taxa are shown in bold. “–” refers to unavailable data; “*” refers to taxa only used in the combined cpDNA dataset; “†” refers to taxa only used in the nrITS dataset.

Taxon	*matK*	Voucher	*rbc*L	Voucher	*rps*16	Voucher	*trn*L-F	Voucher	nrITS	Voucher
*Dodartia orientalis*	MK392230	XZ-2008-1 (KUN)	JQ342984	XZ-2008-1 (KUN)	JQ342982	XZ-2008-1 (KUN)	JQ342981	XZ-2008-1 (KUN)	JQ342980	XZ-2008-1
*Lancea tibetica*	MF786907	Tibet-MacArthur2276 (US)	MF786661	Tibet-MacArthur2276 (US)	FJ172699	XZ-2007-0525 (PE)	FJ172685	XZ-2007-0525 (PE)	FJ172736	XZ-2007-0525
*Mazus alpinus*	MK266256	Sunhang11307 (KUN)	KX783481	Sunhang11307 (KUN)	KX783501	Sunhang11307 (KUN)	KX783520	Sunhang11307 (KUN)	MK192641	Sunhang11307 (KUN)
*Mazus caducifer*	MK266277	kifir037 (KUN)	KX783477	kifir037 (KUN)	KX783497	kifir037 (KUN)	KX783516	kifir037 (KUN)	MK192664	kifir037 (KUN)
Mazus celsioides*	–	–	KX783486	YIF0093 (KUN)	MK266366	YIF0093 (KUN)	KX783525	YIF0093 (KUN)	–	–
*Mazus danxiaensis*	ON323563	CB06425 (CSH)	ON323565	CB06425 (CSH)	ON323567	CB06425 (CSH)	ON323569	CB06425 (CSH)	ON286711	CB06425 (CSH)
*Mazus fauriei*	MK266255	Sunhang11248 (KUN)	–	–	KX783499	Sunhang11248 (KUN)	MK266420	Sunhang11248 (KUN)	LC034207	H. Umemoto: HUP97 (TNS)
*Mazus fruticosus*	MK266261	zdg4447 (KUN)	KX783470	zdg4447 (KUN)	KX783490	zdg4447 (KUN)	KX783509	zdg4447 (KUN)	MK192660	zdg4447 (KUN)
*Mazus gracilis*	–	–	FJ172729	XZ-2007-058 (PE)	FJ172701	XZ-2007-058 (PE)	FJ172687	XZ-2007-058 (PE)	FJ172738	XZ-2007-058 (PE)
Mazus humilis*	–	–	–	–	MK266367	dt804 (KUN)	MK266421	dt804 (KUN)	–	–
*Mazus humilis*†	–	–	–	–	–	–	–	–	MK192667	dt805 (KUN)
*Mazus longipes*	MK266267	Deng1941 (KUN)	KX783474	Deng1941 (KUN)	KX783494	Deng1941 (KUN)	KX783513	Deng1941 (KUN)	MK192652	Deng1941 (KUN)
*Mazus miquelii*	MK266272	NK11186 (KUN)	KX783483	NK11186 (KUN)	KX783503	NK11186 (KUN)	KX783522	NK11186 (KUN)	MK192656	NK11186 (KUN)
*Mazus novaezeelandiae*	MK266278	dtA68 (KUN)	KX783469	dtA68 (KUN)	KX783489	dtA68 (KUN)	KX783508	dtA68 (KUN)	MK192676	dtA68 (KUN)
*Mazus omeiensis*	MK266252	nie1976 (KUN)	KX807209	nie1976 (KUN)	KX807203	nie1976 (KUN)	KX807208	nie1976 (KUN)	MK192636	nie1976 (KUN)
*Mazus procumbens*	MK266261	zdg6074 (KUN)	KX783478	zdg6074 (KUN)	KX783498	zdg6074 (KUN)	KX783517	zdg6074 (KUN)	MK192647	zdg6074 (KUN)
*Mazus pulchellus*	–	–	KX783472	Deng2015 (KUN)	KX783492	Deng2015 (KUN)	KX783511	Deng2015 (KUN)	MK192638	dt093 (KUN)
*Mazus pumilus*	MH265198	M835 (–)	MK266346	Deng403 (KUN)	KX807201	Deng403 (KUN)	KX807206	Deng403 (KUN)	MH711724	SN037
*Mazus pumilus* var. delavayi*	MK266257	Sunhang11459 (KUN)	KX783482	Sunhang11459 (KUN)	KX783502	Sunhang11459 (KUN)	KX783521	Sunhang11459 (KUN)	–	–
*Mazus pumilio*	MK266277	Pagest s.n. 2021829 (KUN)	KX783468	Pagest s.n. 2021829 (KUN)	KX783488	Pagest s.n. 2021829 (KUN)	KX783507	Pagest s.n. 2021829 (KUN)	MK192671	Pagest s.n. 2021829 (KUN)
*Mazus radicans*	–	–	KT626738	K.A. Ford 618785 (CHR)	MK266381	Deng417 (KUN)	–	–	MK192635	Deng417 (KUN)
*Mazus spicatus*	MK266251	zdg3778 (KUN)	FJ172730	XZ-2007-0514 (PE)	FJ172703	XZ-2007-0514 (PE)	FJ172689	XZ-2007-0514 (PE)	FJ172740	XZ-2007-0514 (PE)
Mazus surculosus*	–	–	KX783473	0472212 (KUN)	KX783493	0472212 (KUN)	KX783512	0472212 (KUN)	–	–
Mazus sunhangii*	–	–	KX783485	zdg4600 (KUN)	KX783505	zdg4600 (KUN)	KX783524	zdg4600 (KUN)	–	–
***Mazus unguiculatus***	**ON641731**	**LB1093 (HITBC)**	**ON641733**	**LB1093 (HITBC)**	**ON641735**	**LB1093 (HITBC)**	**ON641737**	**LB1093 (HITBC)**	**ON637063**	**LB1093 (HITBC)**
Mazus xiuningensis*	–	–	MK266349	CY108 (KUN)	MK266384	CY108 (KUN)	MK266430	CY108 (KUN)	–	–

## Results

### Morphological comparison

The putative new species exhibited typical morphology of *Mazus*, being small herbs (Fig. 1A) with obovate to obovate-spatulate leaves, winged petioles (Fig. 1F, G), a strongly two-lipped corolla (upper lip two-lobed, lower lip three-lobed; Fig. 1J–M), and a capsule enclosed within the persistent calyx (Fig. 2D–F). Morphologically, *M. unguiculatus* is most similar to *M. spicatus* by having white to pale rusty pubescence (Fig. 1D), leaves with tapering bases and incised-serrate margins (Fig. 1F), and hairy ovaries (Fig. 2D). However, *M. unguiculatus* exhibits weak, pendulous branches and markedly elongated inflorescences (Fig. 1A), a feature not seen in *M. spicatus*. They also differ markedly in corolla features. The upper corolla lobes are emarginate in *M. unguiculatus* (Fig. 1L) but entire in *M. spicatus*. The three lower corolla lobes of *M. unguiculatus* are subequal, each forming a short claw at the base and shallow teeth at the middle of the apex (Fig. 1K, M), whereas those of *M. spicatus* are unequal, with lateral lobes being much larger. *M. unguiculatus* also lacks a typical palate on the lower lip of the corolla (Fig. 1M), which is present in *M. spicatus*.

### Phylogenetic analysis

**Figure 3.**
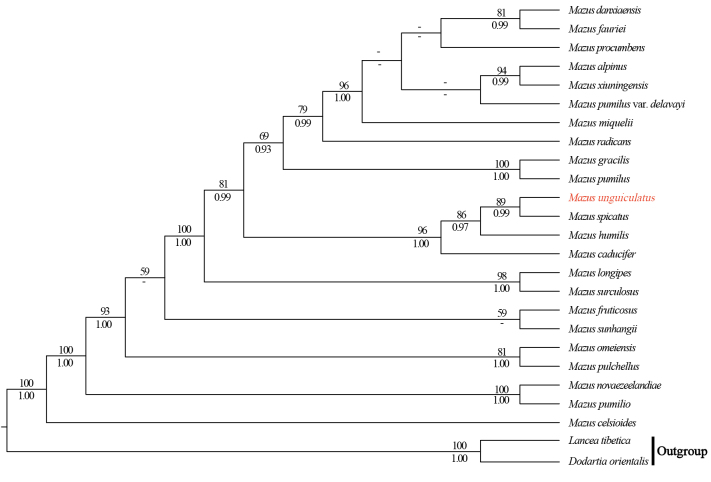
Phylogeny of *Mazus* based on the combined dataset of *matK*, *rbc*L, *rps*16, and *trn*L*-trnF* using the maximum likelihood (ML) method. Bootstrap values (ML-BS) and Bayesian posterior probability values (BI-PP) are displayed above and below the branches, respectively. The new species is highlighted in red. A dash (–) indicates ML-BS < 50% or BI-PP < 0.90.

**Figure 4.**
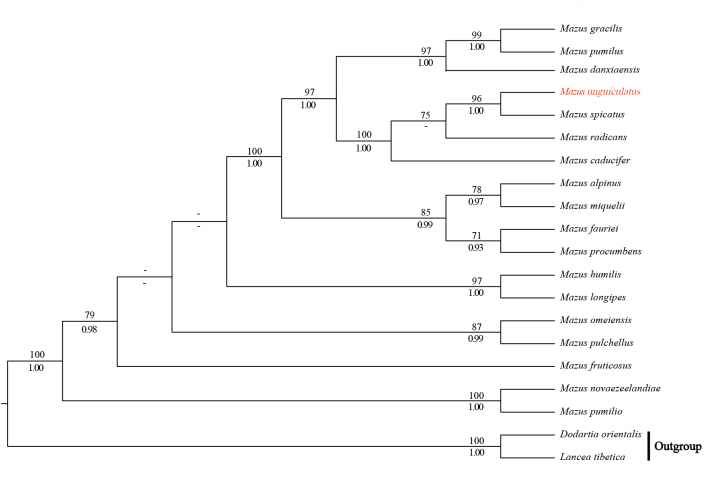
Phylogeny of *Mazus* based on the nrITS dataset using the maximum likelihood (ML) method. Bootstrap values (ML-BS) and Bayesian posterior probability values (BI-PP) are displayed above and below the branches, respectively. The new species is highlighted in red. A dash (–) indicates ML-BS < 50% or BI-PP < 0.90.

The combined cpDNA dataset included 36 aligned sequences with 3,869 characters (858 bp for *rps*16, 855 bp for *matK*, 1,262 bp for *rbc*L, and 894 bp for *trn*L-F). Of these, 328 characters (8.48%) were variable, and 199 characters (5.14%) were parsimony-informative. The nrITS dataset has 30 sequences with an aligned length of 615 bp, of which 169 are variable (27.5%) and 134 are parsimony-informative (21.8%). ML and BI analyses produced highly consistent tree topologies (Figs 3, 4). The ML trees are therefore further discussed below, with the support values of ML bootstrap (ML-BS) and Bayesian posterior probabilities (BI-PP) provided in brackets.

Both cpDNA and nrITS phylogenies strongly supported the monophyly of *Mazus* (Figs 3, 4; ML-BS = 100%, BI-PP = 1.00; all support values follow this order below), resolving a well-supported clade formed by the new species *M. unguiculatus* and *M. spicatus* (cpDNA tree: 89%, 0.99; nrITS tree: 96%, 1.00). However, the placement of this clade within *Mazus* was not consistent. In the cpDNA trees (Fig. 3), the *M. unguiculatus* + *M. spicatus* clade was sister to *M. humilis* Hand.-Mazz. (86%, 0.97) and then to *M. caducifer* Hance (96%, 1.00). In contrast, the nrITS trees (Fig. 4) resolved the clade as sister to *M. radicans* Cheeseman (75%, –), and the three species formed a strongly supported clade with *M. caducifer* (100%, 1.00).

## Discussion

Both morphological and molecular evidence support the distinctness of *M. unguiculatus* and its close relationship with *M. spicatus*. However, its position among several closely related species, i.e., *M. caducifer*, *M. humilis*, and *M. spicatus*, remains unresolved due to persistent topological conflicts, which have already been noted in previous studies (Deng et al. 2019; Li et al. 2022; Chen et al. 2024). *Mazus spicatus* and *M. caducifer* were once assigned to *M.* sect. *Trichogynus* according to their stolons, woody stem bases, and hairy ovaries (Hong et al. 1998; Hsieh 2000). Deng et al. (2019) later found that *M. spicatus* was nested with *M. caducifer* and *M. xiuningensis* X.H.Guo & X.L.Liu, whereas the results of Li et al. (2022) grouped it with *M. humilis* or *M. radicans*. Chen et al. (2024) resolved conflicting relationships of *M. spicatus* with *M. humilis* or the recently discovered *M. jiangshiensis*. This indicates a complex evolutionary history among these taxa, highlighting the need for a broader investigation to cover more recently described species. Nevertheless, the discovery of *M. unguiculatus* marks a further step in unveiling the species diversity of *Mazus* in China, offering new insights into its evolutionary history and ecological adaptation in this region.

### Taxonomic treatment

#### Mazus unguiculatus

Taxon classificationPlantaeLamialesMazaceae

Bo Li sp. nov.

3F515CC8-DA37-5C26-AF54-ED0B5C7ADF38

urn:lsid:ipni.org:names:77379128-1

Figs 1, 2

##### Diagnosis.

This species is most similar to *M. spicatus* in exhibiting white to pale rusty pubescence, leaves with tapering bases and incised-serrate margins, and hairy ovaries. However, it can be readily diagnosed by weak, pendulous branches, a markedly elongated inflorescence, and a distinct corolla, viz., the upper lip is bilobed, each lobe has emarginate to bilobed apex, while the lower lip is deeply trilobed with subequal lobes, each lobe possesses a short claw at the base and shallow teeth at the middle of the apex, and the palate is absent.

##### Type.

China • Anhui Province, Anqing City, Qianshan County, Tianzhushan Mountain, growing epiphytically on the rock wall, 30°42'36.87"N, 116°23'41.24"E, alt. 154 m a.s.l., 27 July 2021, *Xin-Xin Zhu, Jun Wang & Mei-Hua Wang ZXX211303* (holotype HITBC! [barcode HITBC0125448], isotype KUN! [barcode KUN1536783]).

##### Description.

***Herb***, perennial, with white to pale rusty pubescence. Taproot thick, robust, 1.5–4.0 cm long; adventitious roots numerous, shotting from the stem base, slightly woody, dark brown. ***Stem*** many-branched, terete, woody and tuberous at base, pendulous, procumbent, 25–40 cm long; old stems rigid, rooting from lower nodes, brownish green, densely hispidulous to glabrescent; young stems slender, ascending, grayish green, with dense, white hirsuteous hairs. ***Leaves*** sparsely arranged at base, forming a rosette, often caducous; stem leaves numerous, alternate, fascicled at upper nodes; petioles of lower leaves ca. 0.5 cm long, densely hispidulous, gradually reducing in length upward, upper leaves sessile; leaf blade obovate to obovate-spatulate, 0.8–2.4 cm long, 0.6–1.3 cm wide, apex obtuse, base tapering, margin incised-serrate, teeth callous at apex; adaxially green, subglabrous to sparsely hispidulous, abaxially yellowish green, densely hispidulous; veins conspicuous on both surfaces, convex below and slightly concave above. ***Inflorescence*** racemose, many-flowered, terminal and axillary, lax, elongated up to 25 cm long at fruiting; bracts subulate to filiform, 0.6–1.2 mm long; pedicels slender, 0.8–1.4 cm long, with dense, hispidulous hairs and sparse, glandular hairs. ***Calyx*** narrowly campanulate, 5.0–8.0 mm long, 10-veined, densely villous and glandular hairy; lobes 5, triangular-lanceolate, as long as tube, apex acute, ciliate at margin and along veins. ***Corolla*** white to pale violet, 1.1–1.4 cm long, with a darker band on the abaxial surface of upper lip, sparsely puberulent to glabrous; tube cylindric, 0.4–0.6 cm long, exserted from calyx; 2-lipped, upper lip 2-lobed, upcurved, lobes emarginate to slightly 2-lobed; lower lip deeply 3-lobed, lobes subequal, ca. 2.5 × 4.0 mm, obovate, apex round, emarginate or shallowly toothed, base acutely narrowed, forming a short claw; palate absent. ***Stamens*** 4, exserted from throat, inserted at the same level near the apex of the corolla tube; didynamous, anterior 2 longer; separated and opposite to lateral lobes of lower corolla-lip, posterior 2 pairwise approached, included in corolla tube; filaments filiform, curved, glabrous, anther 2-celled, thecae divergent, connivent at apex. ***Style*** 0.6–0.8 cm long, exserted beyond anthers, stigma 2-lamellate; ovary hairy. ***Capsules*** enclosed by the persistent calyx, ca. 0.9 × 1.3 × 2.1 mm, ovoid, compressed, apex rostrated, brown, hispidulous; seeds ovoid, small, numerous.

##### Phenology.

Flowering was observed from June to August and fruiting from July to September.

##### Distribution and habitat.

The species is currently known only from the type locality in Anhui Province, China, specifically in the mountainous regions where it grows on steep rock crevices. The habitat is characterized by a mixed forest community, where the population is found in association with species of *Acer* L., *Pinus* L., and *Miscanthus* Andersson.

##### Etymology.

The specific epithet “unguiculatus” refers to the lobes of the lower corolla lip forming short claws at the base.

##### Vernacular name.

Simplified Chinese: 爪瓣通泉草; Chinese pinyin: Zhuă Bàn Tōng Quán Căo.

##### Provisional conservation status.

*Mazus unguiculatus* is currently known only from a single locality in Anhui Province, China. Given the currently limited survey data, the species is provisionally assessed as Data Deficient (DD) according to the IUCN Red List Categories and Criteria (IUCN 2022). Further surveys are needed to clarify its population size, distribution, and potential threats.

## Supplementary Material

XML Treatment for Mazus unguiculatus

## Additional information

### Artificial Intelligence (AI) use

The authors accept full responsibility for the content of the manuscript, including the disclosure of any use of AI.

No AI tools were used in the preparation of this manuscript.

### Author ORCIDs

S.-C. Xu https://orcid.org/0009-0009-1356-9929

D.-Z. Min https://orcid.org/0000-0002-8779-5592

B. Li https://orcid.org/0000-0003-1628-8128

### Data availability

All of the data that support the findings of this study are available in the main text.
